# A genomic resource for a hidden endemic: the complete chloroplast genome of *Allium aktauense* from Uzbekistan

**DOI:** 10.12688/f1000research.184132.3

**Published:** 2026-10-28

**Authors:** Ibrokhimjon Ergashov, Farkhodjon Mingboev, Ziyoviddin Yusupov, Farruhbek Rasulov, Diyorjon Hamrayev, Mashrab Turdiyev, Gulirukhsor Ganiyeva, Movlon Bekmirzayev, Yulduz Fayzulloyeva, Saidkamol Xaydarov, Shohruh Fayzulloyev, Zera Moldakhmetova, Laylo Mirzoyeva, Dilbar Esankulova

**Affiliations:** 1 Institute of Botany, Academy of Sciences of Uzbekistan, Tashkent, Uzbekistan; 2 2Department of Pharmaceutical Sciences, Andijan State Medical Institute, Andijan, Uzbekistan; 3 Bukhara State Medical Institute, Bukhara, Uzbekistan; 4 Termez State University, Surkhandarya, Uzbekistan; 5 Jizzakh State Pedagogical University, Jizzakh, Uzbekistan; 6 Bukhara State Pedagogical Institute, Bukhara, Uzbekistan; 7 Department of Botany and Biotechnology, Fergana State University,, Fergana, Uzbekistan; 8 Bukhara State University, Bukhara, Uzbekistan; 9 Karakalpak State University, Nukus, Karakalpakstan, Uzbekistan; 10 Termez State Pedagogical Institute, Surkhandarya, Uzbekistan; 11 Jizzkah State Pedagogical University, Jizzkah, Uzbekistan

**Keywords:** Allium aktauense; chloroplast genome; phylogenomics; endemic species; Central Asia; conservation genetics.

## Abstract

*Allium aktauense* is a narrow endemic species from the Aktau Range in the Nurotau Mountains, Western Pamir-Alay, Uzbekistan, where it grows in rocky limestone crevices. Genomic resources for this species have previously been limited, restricting molecular assessment of its phylogenetic placement. In this study, we sequenced, assembled and characterized the complete chloroplast genome of *A. aktauense* and evaluated its phylogenetic position within *Allium* using comparative plastome data. The plastome is 152,443 bp long and has the typical quadripartite organization, comprising a large single-copy region of 81,522 bp, a small single-copy region of 17,907 bp and two inverted repeat regions of 26,507 bp each. Annotation identified 131 gene copies, including 85 protein-coding genes, 38 tRNA genes and 8 rRNA genes. Across the broad comparative dataset, the highest nucleotide diversity was observed in the *ndhF-rpl32* region (Pi approximately 0.044), followed by *ycf1* (Pi approaching 0.030); these regions are treated here as candidate variable loci requiring further validation within closely related taxa. Maximum-likelihood analysis of complete plastome sequences placed *A. aktauense* within subgenus *Allium*, with *A. ophiophyllum* as its closest sampled plastome lineage (bootstrap support 100%). The plastome provides a genomic resource for future phylogenetic, taxonomic and conservation-related research on endemic Central Asian *Allium*.

## Introduction

The genus *Allium* L. is one of the largest and taxonomically most complex genera of monocotyledons, comprising more than 1000 species distributed mainly across temperate and seasonally dry regions of the Northern Hemisphere ( Friesen et al., 2006, Li et al., 2010). The genus includes economically important vegetable crops, medicinal plants and ornamentals, and is traditionally recognized by bulbous or rhizomatous life forms, membranous to fibrous bulb tunics, free or nearly free tepals and a characteristic sulphur-containing odour. Southwestern and Central Asia, together with the Mediterranean region, represent one of the principal centers of diversity and diversification for *Allium*, whereas a second center occurs in western North America ( Friesen et al., 2006; Nguyen et al., 2008; Li et al., 2010; Khassanov, 2018; Jang et al., 2023).

Central Asia is especially important for understanding the evolution of *Allium*, because the region harbours numerous narrowly distributed and locally endemic taxa associated with mountain systems, rocky slopes, gypsum- and limestone-rich substrates, and arid to semi-arid habitats ( Khassanov and Yussupov 2022, Ergashov et al. 2025a). Recent taxonomic, morphological and molecular studies have shown that species boundaries and infrageneric relationships in *Allium* are often difficult to resolve using morphology alone, owing to high morphological diversity, convergent ecological adaptations and incongruence between traditional sectional classifications and molecular phylogenies ( Friesen et al., 2006; Li et al., 2010; Yusupov et al., 2022; Jang et al., 2023; Ergashov et al., 2026b). Complete plastome data therefore provide a complementary source of evidence for phylogenetic placement and for identifying variable genomic regions.

*Allium aktauense* F.O.Khass. & Esankulov is a narrow endemic species described from the Aktau Range in the Western Pamir-Alay ( Khassanov and Esankulov, 2015). The species was originally placed in *Allium* subgen. *Allium* sect. *Minuta* F.O.Khass., a small Middle Asian section characterized by diminutive plants, a compressed perianth and persistent spathe ( Figure 1) ( Khassanov and Yussupov 2022). Its extremely restricted distribution and occurrence in rocky limestone crevices make it a valuable species for studies of local endemism, habitat specialization and conservation genetics in the Western Pamir-Alay ( Beshko et al., 2025).

**Figure 1.**
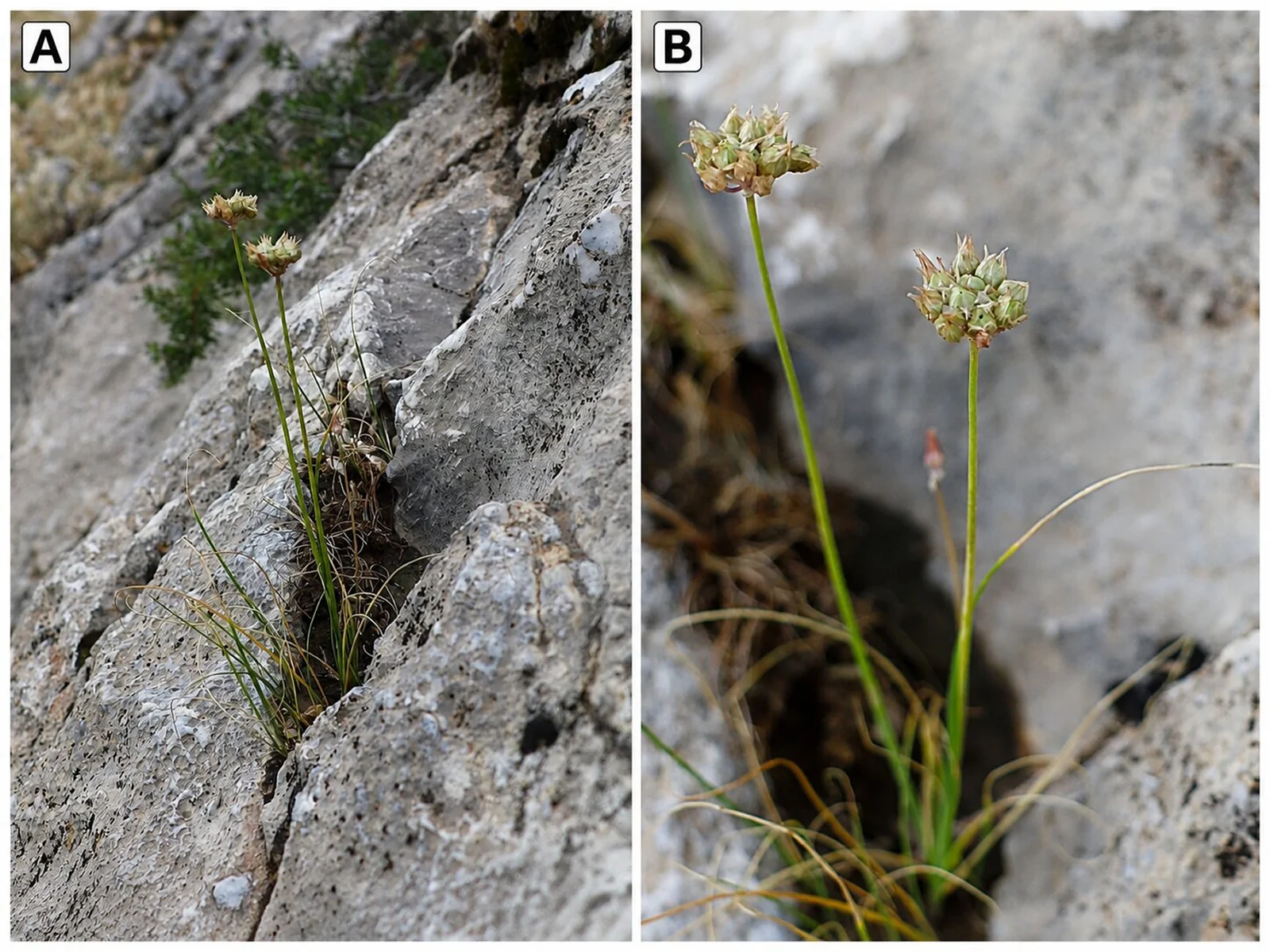
Habitat and morphology of *Allium aktauense* in its type locality, northern slopes of Lyangar Pass, Aktau Range, Nurotau Mountains, Western Pamir-Alay, Uzbekistan (40°20′37.08″ N, 66°00′34.69″ E). A, general view of plants growing in rocky limestone crevices; B, close-up view of the inflorescences. Photograph taken by N. Beshko.

Chloroplast genomes have become widely used in plant systematics because of their generally conserved quadripartite structure, uniparental inheritance, moderate evolutionary rate and usefulness for reconstructing phylogenetic relationships at interspecific and infrageneric levels. In *Allium*, plastome-scale studies have improved understanding of phylogenetic relationships, divergence patterns, adaptive evolution and the utility of hypervariable regions as candidate molecular markers ( Xie et al., 2020; Chen et al., 2022; Yang et al., 2023). Despite increasing plastome sampling across *Allium*, genomic information for many narrowly distributed Central Asian endemics remains limited. This is particularly true for species of small, geographically restricted sections such as sect. *Minuta*, for which molecular data are still insufficient to test morphology-based classifications and to evaluate relationships with other lineages of subg. *Allium.* Generating the complete chloroplast genome of *A. aktauense* is therefore important not only for documenting the genomic resources of a narrow endemic species, but also for improving comparative plastome sampling in Central Asian *Allium.*

In the present study, we sequenced, assembled and annotated the complete chloroplast genome of *A. aktauense* collected from its type region in the Aktau Range, Western Pamir-Alay, Uzbekistan. We aimed to: (1) characterize the overall structure, gene content and organization of the chloroplast genome; (2) identify highly variable regions that may be useful as molecular markers; (3) infer its phylogenetic position within the genus using complete chloroplast genome data. This study provides the first plastome resource for *A. aktauense* and contributes to ongoing efforts to clarify the evolution, taxonomy and conservation value of endemic *Allium* species of Central Asia.

## Materials and methods

Fresh leaf material of *Allium aktauense* was sampled in May 2025 from a natural population at the type locality on the northern slopes of Lyangar Pass, Aktau Range, Nurotau Mountains, Western Pamir-Alay, Uzbekistan (40°20′37.08″ N, 66°00′34.69″ E; Figure 1). Species identification was carried out by Ibrokhimjon Ergashov at the National Herbarium of Uzbekistan, and representative voucher specimens were deposited in the National Herbarium of Uzbekistan (TASH) under voucher accession number TASH0043. Leaf samples collected for molecular work were rapidly desiccated in silica gel in the field and subsequently kept at room temperature until DNA isolation.

Genomic DNA was isolated from the dried leaf tissue using the Tiangen plant genomic DNA extraction kit according to the supplier’s instructions. The overall plastome sequencing and bioinformatic workflow followed standard procedures used in recent chloroplast genome studies, with minor modifications appropriate for *Allium* material ( Ergashov et al., 2026a). Illumina sequencing libraries were constructed using a NEB library preparation kit. Genomic DNA was sheared to an average insert size of approximately 350 bp, followed by end repair, adapter ligation, and PCR enrichment. Library integrity and fragment-size distribution were evaluated using an Agilent 5400 system, and the final library concentration was quantified before sequencing. Qualified libraries were sequenced on an Illumina platform by Novogene Bioinformatics Technology Co.

Clean sequencing reads supplied by Novogene Bioinformatics Technology Co. were used for de novo chloroplast genome assembly with NOVOPlasty 4.3.1 ( Dierckxsens et al., 2017) under default assembly settings. The original unfiltered sequencing reads were not supplied to the authors. The assembly yielded a complete chloroplast genome sequence of 152,443 bp. Genome annotation was carried out in Geneious Prime v2025.1.2, using the chloroplast genome of *Allium nikolaii* F.O.Khass. & Achilova (NC_068793) as a reference. The preliminary annotation was inspected manually, and gene boundaries were corrected where necessary, including verification of start and stop codons as well as exon–intron junctions in protein-coding genes ( Kearse et al., 2012). The circular plastome map was generated with CPGView (Liu et al., 2023; Figure 2).

**Figure 2.**
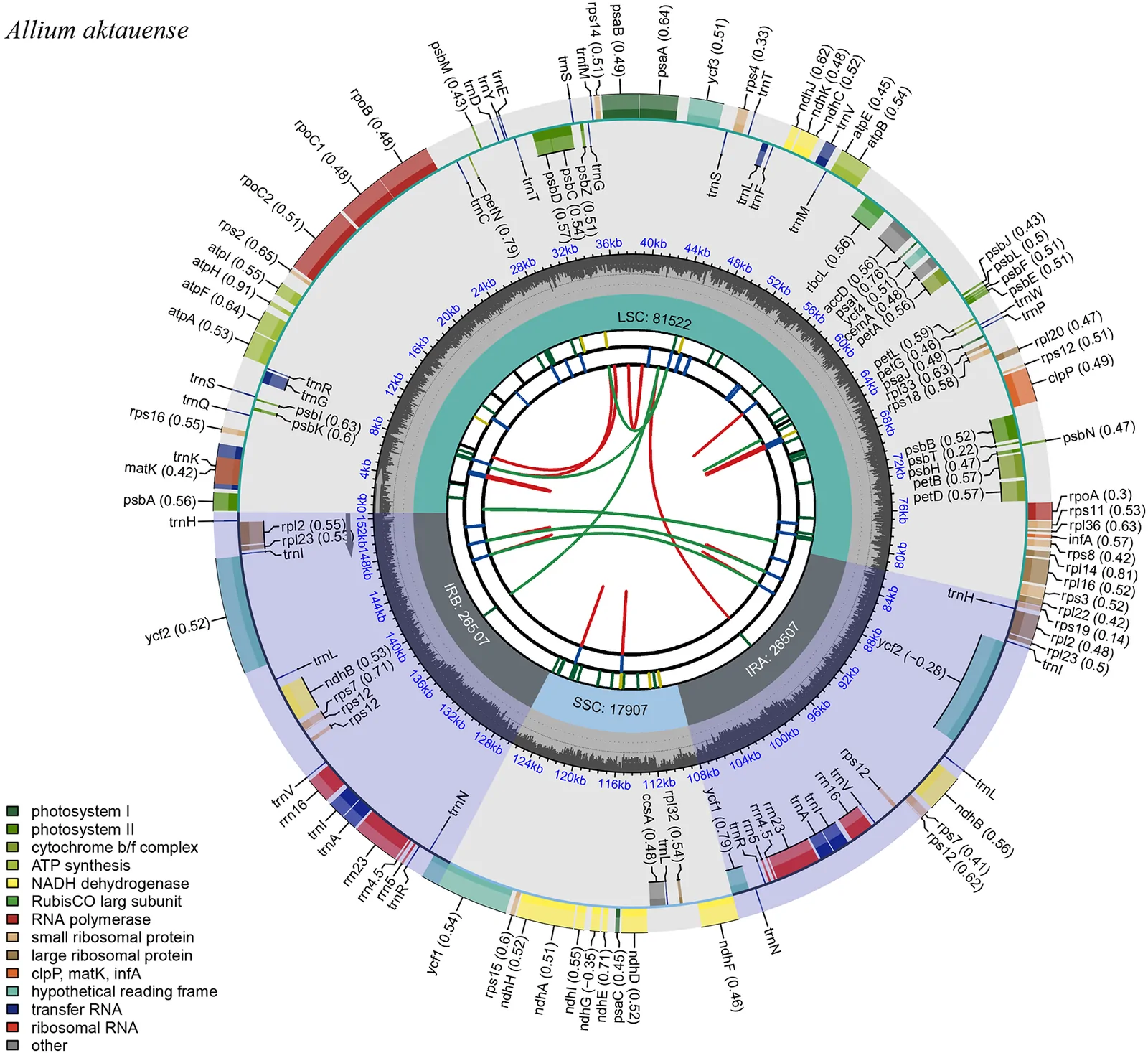
Circular map of the complete chloroplast genome of *Allium aktauense.* From the center outward, the map consists of six concentric rings. The innermost ring shows forward and reverse repeats, connected by red and green arcs, respectively. The second ring indicates tandem repeats, while the third ring displays microsatellite sequences. The fourth ring shows the sizes of the large single-copy (LSC), small single-copy (SSC), and two inverted repeat regions (IRa and IRb). The fifth ring represents the distribution of GC content. The outermost ring displays genes grouped according to their functional categories with the corresponding codon usage bias values shown in parentheses after each gene name.

For comparative plastome analyses, complete chloroplast genome sequences of representative *Allium* species were downloaded from NCBI GenBank ( Table 1). The plastome sequences were aligned using MAFFT v7.5 ( Katoh and Standley, 2013) no IR copy was removed before the alignment described here. Nucleotide diversity (Pi) was estimated in DnaSP v6.12.03 ( Rozas et al., 2017) using a sliding-window approach with a window size of 800 bp and a step size of 200 bp. Because the dataset spans multiple subgenera, highly variable windows identified in this analysis are interpreted as candidate regions at this broad interspecific sampling scale and not as validated population-level markers for sect. *Minuta*.

**Table 1.** NCBI accession numbers of the species of the genus *Allium* and outgroups.

N	Species name	NCBI accession number
1.	*Agapanthus coddii*	MT348439
2.	*Narcissus poeticus*	NC_039825
3.	*Allium ampeloprasum*	OR601505
4.	*Allium tianschanicum*	PZ015921
5.	*Allium teretifolium*	NC_080287
6.	*Allium spurium*	NC_077660
7.	*Allium splendens*	NC_084237
8.	*Allium spirale*	NC_068826
9.	*Allium senescens*	NC_057580
10.	*Allium schoenoprasum*	NC_057575
11.	*Allium sativum*	OR605582
12.	*Allium sacculiferum*	NC_070336
13.	*Allium roylei*	NC_084236
14.	*Allium pskemense*	OR605578
15.	*Allium pseudojaponicum*	NC_080286
16.	*Allium prostratum*	NC_077659
17.	*Allium praemixtum*	NC_044412
18.	*Allium oschaninii*	OR605577
19.	*Allium ophiophyllum*	PZ269957
20.	*Allium nutans*	PZ015918
21.	*Allium nikolaii*	NC_068793
22.	*Allium montanostepposum*	PZ015917
23.	*Allium mongolicum*	ON008504
24.	*Allium minus*	OQ701011
25.	*Allium lusitanicum*	OR884983
26.	*Allium longistylum*	NC_080285
27.	*Allium lineare*	PZ015916
28.	*Allium ledebourianum*	PV684023
29.	*Allium koreanum*	NC_057579
30.	*Allium hymenorrhizum*	NC_084234
31.	*Allium herderianum*	NC_042156
32.	*Allium glaciale*	NC_084233
33.	*Allium galanthum*	NC_050981
34.	*Allium fuscoviolaceum*	NC_084232
35.	*Allium forrestii*	NC_049101
36.	*Allium flavescens*	NC_077655
37.	*Allium fistulosum*	NC_040222
38.	*Allium filidens*	NC_068789
39.	*Allium dumebuchum*	NC_077654
40.	*Allium cretaceum*	PZ015913
41.	*Allium condensatum*	OP743932
42.	*Allium cernuum*	OR605569
43.	*Allium caricoides*	NC_062825
44.	*Allium caespitosum*	NC_068828
45.	*Allium austrosibiricum*	NC_077653
46.	*Allium atrosanguineum*	PZ015912
47.	*Allium angulosum*	NC_077652
48.	*Allium amphibolum*	NC_084231
49.	*Allium altaicum*	NC_040972
50.	*Allium cyathophorum* var. *farreri*	MK931245
51.	***Allium aktauense***	**QB045826**

Phylogenetic relationships among the sampled *Allium* species were reconstructed using complete chloroplast genome sequences. The aligned plastome matrix generated in MAFFT v7.5 was analyzed under a maximum likelihood (ML) framework in RAxML v8.2.12 ( Stamatakis, 2014), applying the GTRGAMMA substitution model. Branch support was assessed with 1,000 bootstrap replicates. The resulting tree was rooted with *Agapanthus coddii* F.M.Leight. and *Narcissus poeticus* L., representing related genera within Asparagales, to provide an external comparative framework for assessing the phylogenetic placement of *A. aktauense* within *Allium.*

## Results

The complete chloroplast genome of *Allium aktauense* showed a typical quadripartite structure, including one large single-copy region, one small single-copy region and two inverted repeat regions ( Figure 2). The chloroplast genome was 152,443 bp in length, including an LSC region of 81,522 bp, an SSC region of 17,907 bp, and two IR regions of 26,507 bp each. Genome annotation identified 131 genes, including 85 protein-coding sequences (CDS), 38 transfer RNA (tRNA) genes, and 8 ribosomal RNA (rRNA) genes. This genome organization is consistent with most angiosperm plastomes and agrees with previously published chloroplast genomes of *Allium* species ( Munavvarov et al. 2022; Ergashov et al. 2025a, b, 2026a, b). The plastome size of *A. aktauense* (152,443 bp) is also close to those reported for other members of the genus, such as *A. wallichii* (152,496 bp) ( Zhao et al., 2023).

Sliding-window analysis of nucleotide diversity (Pi) revealed a heterogeneous distribution of sequence variation across the chloroplast genome alignment ( Figure 3). Several highly variable regions were identified, mainly in intergenic spacers and selected coding regions. The most variable regions included *rps16*, *trnD-GUC–trnE-UUC *, *psbJ–psbE*, *ndhF–rpl32*, and *ycf1.* Among these, the *ndhF–rpl32* region showed the highest nucleotide diversity, with Pi reaching approximately 0.044. The *ycf1* region also showed elevated variation, with Pi values approaching 0.030. These regions may represent candidate variable loci for species identification and phylogenetic reconstruction, but their utility in future population-level studies requires further validation. Among these loci, *ycf1* is particularly notable because it has frequently been reported as one of the most variable and phylogenetically informative plastid regions in angiosperms and in several monocot lineages ( Dong et al. 2012, 2015). In the present study, *ycf1* also showed high nucleotide diversity, supporting its potential value for resolving relationships among closely related *Allium* species ( Figure 3). The *ndhF–rpl32* intergenic region showed the highest variation, suggesting that non-coding plastid regions may contain stronger species-level signals than many conserved coding regions ( Gielly and Taberlet, 1994; Shaw et al. 2007).

**Figure 3.**
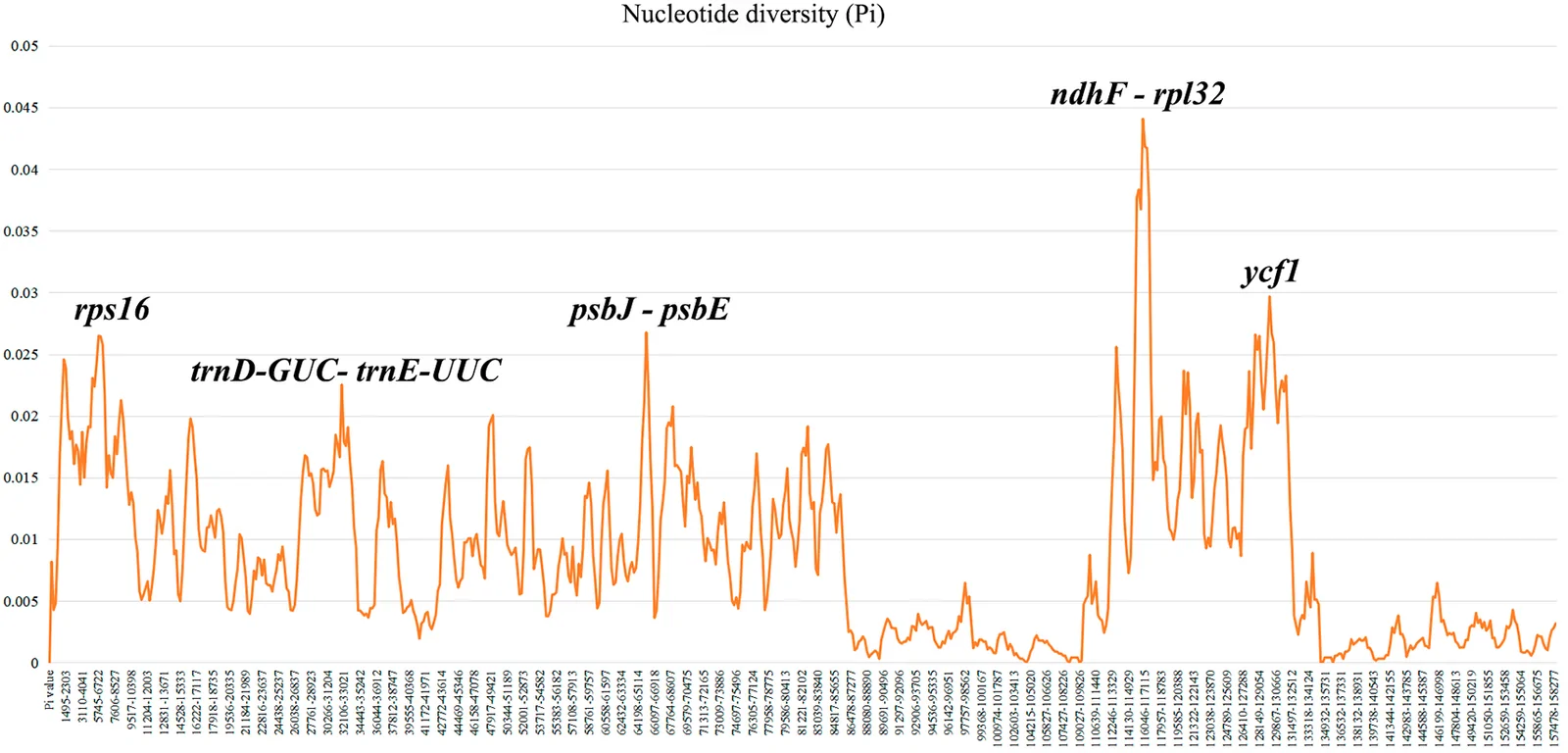
Nucleotide diversity (Pi) across the aligned complete chloroplast genomes of the *Allium* species based on sliding-window analysis. The x-axis shows genome/alignment position in kilobases and the y-axis shows nucleotide diversity (Pi). Window length: 800 bp; step size: 200 bp.

The chloroplast genome phylogeny placed *A. aktauense* within subgenus *Allium*, supporting its current broad taxonomic placement ( Figure 4). The maximum likelihood tree recovered several major clades corresponding to recognized *Allium* lineages, including *Cepa*, *Polyprason*, *Reticulatobulbosa*, *Amerallium*, *Rhizirideum*, and subgenus *Allium.* Bootstrap support values indicated that complete chloroplast genome sequences provided a strong phylogenetic signal for resolving relationships among the sampled taxa. In the plastome tree, *A. aktauense* was recovered close to *A. ophiophyllum*, while *A. nikolaii* and *A. filidens* formed a neighbouring lineage. However, this result should be interpreted cautiously. The close position of *A. aktauense* to *A. ophiophyllum* in the plastome tree does not necessarily mean that these species are closest relatives in a complete evolutionary sense. Chloroplast genomes represent a maternally inherited or uniparentally inherited history in many angiosperms and may differ from nuclear phylogenetic patterns, especially in groups affected by hybridization, incomplete lineage sorting or rapid radiation ( Folk et al. 2017; Ye et al. 2021). Therefore, the plastome tree provides strong evidence for the placement of *A. aktauense* within subgenus *Allium*, but broader sampling of sect. *Minuta* and related Middle Asian sections, together with nuclear markers, will be necessary to clarify its precise sectional relationships.

**Figure 4.**
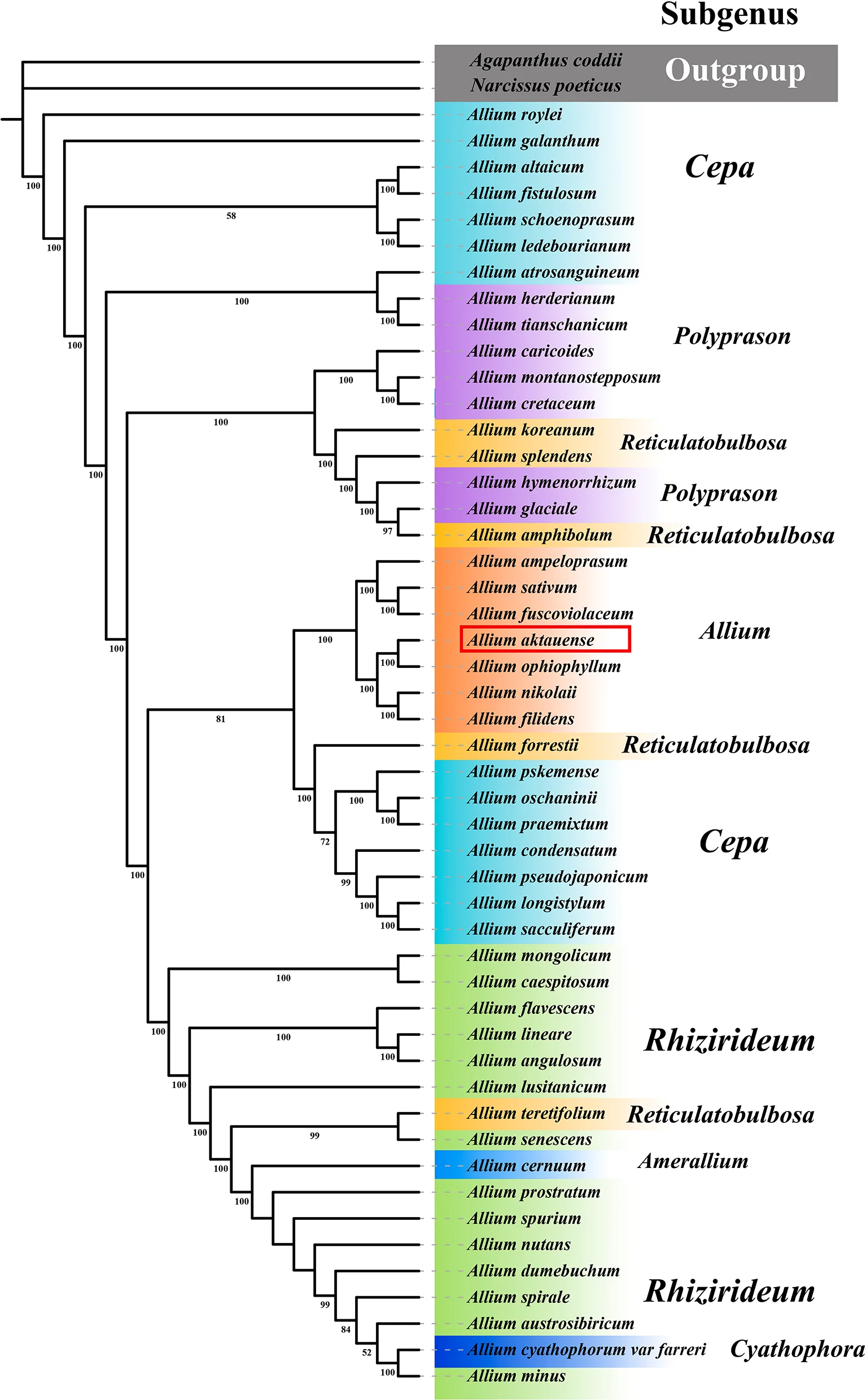
Maximum-likelihood phylogenetic tree of the *Allium* species inferred from complete chloroplast genome sequences. *Allium aktauense* is highlighted with a red box. Numbers at nodes are bootstrap support percentages based on 1,000 replicates. Black branches are the graphical branches of the tree and coloured background bands indicate taxonomic assignments. Branch colours indicate bootstrap support values.

Overall, the present study provides the first complete chloroplast genome of *A. aktauense* and adds an important genomic resource for Central Asian *Allium.* The conserved plastome structure, identification of highly variable loci and phylogenetic placement within subgenus *Allium* contribute to a better understanding of the taxonomy, evolution and conservation value of this narrow endemic species.

## Software availability

No custom code was used in this study.

Software used in the analysis included NOVOPlasty, Geneious Prime, DnaSP v6.12.03, MAFFT, RAxML as cited in the Methods section.

## Data Availability

NCBI GenBank: *Allium aktauense* chloroplast genome. Accession number QB045826. The accession numbers of comparative chloroplast genomes used in this study are provided in Table 1. NCBI BioProject: Illumina sequencing data associated with *Allium aktauense*. BioProject accession number: PRJNA1534234. NCBI BioSample: *Allium aktauense* sample associated with the sequencing data. BioSample accession number: SAMN63435527. NCBI: Sequence Read Archive (SRA): Illumina sequencing reads of *Allium aktauense*. SRA accession number: SRR40827210.
