# Supplementary material for: A genomic resource for a hidden endemic: the complete chloroplast genome of Allium aktauense from Uzbekistan
Source: F1000Res. 2026 Oct 28;15:1296. Originally published 2026 Aug 5. [Version 3] doi: 10.12688/f1000research.184132.3 (PMC13731900; doi:10.12688/f1000research.184132.3)
Supplement: Peer review history

# Reviewer report: Reviewer response for version 3

**DOI:** 10.5256/f1000research.213480.r534830

**Published:** 2026-11-03

**Authors:** Hoang Dang Khoa Do

**Affiliations:** 1 Nguyen Tat Thanh University, Ho Chi Minh City, Ho Chi Minh, Vietnam

Thank you very much for revising the manuscript based on the reviewer's comments.

 I do not have more comments on the revised manuscript.

Are the datasets clearly presented in a usable and accessible format, and the assembly and annotation available in an appropriate subject-specific repository?

Yes

Are sufficient details of the sequencing and extraction, software used, and materials provided to allow replication by others?

Yes

Are the rationale for sequencing the genome and the species significance clearly described?

Yes

Are the protocols appropriate and is the work technically sound?

Yes

Reviewer Expertise:

Plant Genomics, chloroplast genome evolution.

I confirm that I have read this submission and believe that I have an appropriate level of expertise to confirm that it is of an acceptable scientific standard.

# Reviewer report: Reviewer response for version 2

**DOI:** 10.5256/f1000research.211682.r530316

**Published:** 2026-10-23

**Authors:** Ruslan Kalendar

**Affiliations:** 1 University of Helsinki, Helsinki, Finland

I thank the authors for their revision. The main problems raised on version 1 have been addressed:

 •    The plastome has been re-deposited under QB045826, and its length now matches the manuscript (152,443 bp).

 •    The Illumina reads are available in SRA (PRJNA1534234 / SAMN63435527 / SRR40827210).

 •    Table 1 and Figure 4 now contain the same 51 terminals.

 •    Numerical bootstrap values are shown in Figure 4, and the key A. aktauense-A. ophiophyllum node is reported with 100% support.

 •    The non-monophyly of the sampled Cepa and Polyprason representatives is now stated openly.

 •    The handling of the inverted repeats in the Pi analysis is clarified, and the marker claims are framed as candidate loci.

 The article now meets the standard expected of a Genome Note, and I am pleased to approve it.

 Several changes described in the authors’ response do not yet appear in the published text of version 2. None requires new analyses, and I ask that they be corrected in the next version:

 1.    Table 1 (entry 51) and the Data availability section still cite PX504239. Please replace it with QB045826 and ensure that the record is publicly released, as Dr Do also requested.

 2.    The paragraph beginning “The chloroplast genome phylogeny placed A. aktauense within subgenus Allium…” is carried over from version 1. It still states that the tree recovered major clades corresponding to recognized Allium lineages, which contradicts the new paragraph before it. It also repeats the plastid-inheritance caveat almost verbatim. Please delete it. Likewise, the size comparison with A. wallichii and the phrase “conserved plastome structure” in the final paragraph remain, although the response states they were removed.

 3.    Figure 4 legend. The last sentence, “Branch colours indicate bootstrap support values”, contradicts the preceding sentence and should be deleted.

 4.    “Conservation genetics” is still listed as a keyword. Please replace it with “plastome”, as stated in the response. A one-sentence statement on collecting permits and compliance with national access regulations would still be appropriate for a narrow endemic collected at its type locality.

 5.    jModelTest and OGDRAW are still listed, although neither was used; the map was produced with CPGView. NOVOPlasty v4.3.1 and CPGView should be listed instead. Liu et al. (2023) is cited in the text but missing from the reference list. Greiner et al. (2019) is listed but not cited.

 6.   The Grant information states that no grants supported this work, but the Acknowledgment lists three funded projects (the “Digital Nature” State Program, AL-9224104464 and FL-10425067174). Please reconcile these statements.

 7.    In Results, Ergashov et al. (2025b) and Ergashov et al. (2026b) are cited as published Allium chloroplast genomes. However, 2025b is a climate-vulnerability study and 2026b concerns Lepidium olgae. Ergashov et al. (2026b) is also cited in the Introduction in support of a statement about Allium classification. Please check these citations.

Are the datasets clearly presented in a usable and accessible format, and the assembly and annotation available in an appropriate subject-specific repository?

Partly

Are sufficient details of the sequencing and extraction, software used, and materials provided to allow replication by others?

Partly

Are the rationale for sequencing the genome and the species significance clearly described?

Yes

Are the protocols appropriate and is the work technically sound?

Partly

Reviewer Expertise:

systematics, comparative analysis

I confirm that I have read this submission and believe that I have an appropriate level of expertise to confirm that it is of an acceptable scientific standard.

# Reviewer report: Reviewer response for version 2

**DOI:** 10.5256/f1000research.211682.r530317

**Published:** 2026-10-20

**Authors:** Hoang Dang Khoa Do

**Affiliations:** 1 Nguyen Tat Thanh University, Ho Chi Minh City, Ho Chi Minh, Vietnam

Thank you very much for revising the manuscript based on the reviewer's comments.

 Please check my comments for minor issues below.

 1/ In Table 1 and Data availability, please replace old accession number of Allium (PX504239) to the new one (QB045826).

 2/ Please ask GenBank staff to release QB045826 for public access.

Are the datasets clearly presented in a usable and accessible format, and the assembly and annotation available in an appropriate subject-specific repository?

Yes

Are sufficient details of the sequencing and extraction, software used, and materials provided to allow replication by others?

Yes

Are the rationale for sequencing the genome and the species significance clearly described?

Yes

Are the protocols appropriate and is the work technically sound?

Yes

Reviewer Expertise:

Plant Genomics, chloroplast genome evolution.

I confirm that I have read this submission and believe that I have an appropriate level of expertise to confirm that it is of an acceptable scientific standard, however I have significant reservations, as outlined above.

# Response

**Published:** 2026-10-23

**Authors:** Ibrokhimjon Ergashov

**Affiliations:** Botany, Institute of Botany of the Academy of Sciences of the Republic of Uzbekistan, Tashkent, Tashkent Province, Uzbekistan

Thank you very much for your helpful comments and suggestions.

1. We have replaced the old GenBank accession number (PX504239) with the new accession number (QB045826) in Table 1 and the Data Availability section.
2. We have contacted GenBank staff and requested that accession QB045826 be released for public access.

# Reviewer report: Reviewer response for version 1

**DOI:** 10.5256/f1000research.203248.r509506

**Published:** 2026-08-17

**Authors:** Ruslan Kalendar

**Affiliations:** 1 University of Helsinki, Helsinki, Finland

This genome note reports the first complete plastome of Allium aktauense F.O.Khass. and Esankulov, a narrow endemic of the Aktau Range (Nurotau Mountains, Western Pamir-Alay, Uzbekistan), assigned to Allium subgen. Allium sect. Minuta. The authors assembled a 152,443 bp quadripartite plastome, annotated 131 genes, screened 50 aligned plastomes for nucleotide diversity, and inferred a maximum-likelihood tree placing A. aktauense within subgen. Allium, close to A. ophiophyllum.

 The species is genuinely under-sampled, and the addition of a sect. Minuta plastome to the comparative pool is a worthwhile contribution. Central Asian endemics are where plastome sampling in Allium remains thinnest. The collection is properly vouchered (TASH0043) at the type locality, the software chain is conventional and appropriate, and the authors are commendably cautious in their closing paragraph about not over-reading a plastid-only topology.

 However, several problems must be resolved before the record can be considered reliable. Two are serious: 

 (i) the deposited GenBank record is flagged UNVERIFIED, and its stated length does not match the manuscript, so the primary deliverable of the paper is currently not usable as published; 

 and (ii) Figure 4 and Table 1 do not describe the same taxon set, which means that the phylogenetic analysis presented cannot be reproduced from the information given. 

 Beyond these, the paper reports substantially less about the assembly and the plastome itself than is standard for a Genome Note, and several claims in the Results are asserted rather than demonstrated.

 Major comments

 1. This is a central issue. The manuscript reports 152,443 bp, and the partition sizes given (LSC 81,522 + SSC 17,907 + 2 × IR 26,507) sum exactly to 152,443. The deposited record, however, is 152,433 bp, a 10 bp difference. Because the manuscript's own arithmetic is self-consistent, this is not a typographical slip in one of the partition values; it implies that the sequence described in the text is not the sequence deposited or that the deposit was revised after the manuscript was written. The authors need to determine which is correct, correct the text and/or resubmit the record, and explicitly state in the revision which version is definitive.

 The UNVERIFIED flag compounds this. In GenBank, this flag typically signals problems detected in the annotation, most often internal stop codons, frameshifted CDS, or protein products that fail similarity screening. Since the entire value of a Genome Note rests on the deposited record being correct and usable, the authors must resolve the flag before this version can be approved. Concretely, I would suggest:

 •    Re-annotate independently of the single-reference approach (see comment 5) and cross-check every CDS for start/stop codons and the correct reading frame.

 •    Verify tRNA predictions using a dedicated tool (tRNAscan-SE or ARAGORN) rather than by transfer from a reference.

 •    Confirm the IR boundaries and IR sequence identity, as junction errors are a common source of length discrepancies of this magnitude.

 2. The Methods section describes library preparation and states that NOVOPlasty was used; however, the manuscript provides no quantitative account of the sequencing or assembly. There is no figure for total raw reads, clean reads after filtering, trimming tool and parameters used to produce those "high-quality clean reads,” proportion of reads mapping to the plastome, mean or minimum coverage depth, seed sequence used to initiate the NOVOPlasty assembly, or k-mer setting. Nor is it stated whether the assembly closed as a single circular contig or required manual joining, and whether the junctions were validated (by PCR or by read-depth continuity across the IR/SC boundaries).

 For a Genome Note, this is a significant omission, as these numbers are the primary evidence that the assembly is trustworthy, and their absence is particularly consequential given comment 1. Please add a short paragraph or a small table reporting these values. 

 3. The Data availability section lists only the assembled plastome. The underlying Illumina reads should be deposited in the SRA under a registered BioProject and BioSample, with the accessions cited. This is a requirement, independent of journal policy: without the reads, no one can re-assemble or re-annotate the plastome, which is precisely what the UNVERIFIED status now makes necessary. I would treat this as a requirement rather than a suggestion.

 Similarly, the MAFFT alignment matrix and the RAxML tree file (with support values) should be deposited in a repository such as Figshare, Zenodo, Dryad, or TreeBASE and cited. At present, neither the diversity analysis nor the phylogeny can be reproduced from what is provided.

 4. Table 1 lists 50 accessions (48 Allium plus two outgroups, including A. aktauense). Figure 4 shows the 51 terminals. They are checked against each other.

 •    Present in the tree but absent from Table 1, with no accession number given anywhere: Allium ampeloprasum, Allium cernuum, and Allium cyathophorum var. farreri.

 •    Present in Table 1 but absent from the tree: Allium vavilovii (NC_084238) and Allium cepa (NC_024813).

 This needs to be reconciled. As it stands, the analysis cannot be repeated, and the omission of A. cepa in particular is conspicuous given that the Cepa clade is labelled in the figure. Please make Table 1 the complete and exact list of sequences used, and confirm that the tree was built from that same matrix.

 While revising Table 1, please also confirm that all accessions are publicly released. Several (PZ015912–PZ015921, PZ269957) share a prefix suggesting very recent submission, and if any are still under embargo, the comparative analysis is not currently reproducible.

 5. The Results state that the ML tree "recovered several major clades corresponding to recognized Allium lineages, including Cepa, Polyprason, Reticulatobulbosa, Amerallium, Rhizirideum, and subgenus Allium." The figure does not show this. As labelled, Cepa appears in at least three separate positions, Polyprason in two, Reticulatobulbosa in two, and Amerallium in two widely separated places with A. cernuum labelled Amerallium while nested inside the Rhizirideum clade, and A. cretaceum labelled Amerallium in the middle of an otherwise unrelated grade. Either the subgenera are strongly non-monophyletic in this plastome tree, which is an interesting result but is not what the text says and is not discussed, or the annotation bands in the figure are misplaced.

 Please check the labelling carefully. If non-monophyly is real, it should be stated plainly and discussed (plastid capture and incomplete lineage sorting have already been raised in the Discussion and would be the natural framing). If it is a figure error, it should be corrected. Either way, the sentence in the Results section needs to be rewritten, because at present, the text asserts a level of concordance with the classification that the figure does not support.

 6. The manuscript states that "Bootstrap support values indicated that complete chloroplast genome sequences provided a strong phylogenetic signal," but no support value appears anywhere in the text, including for the two nodes the paper's conclusions actually rest on: the placement of A. aktauense within subgen. Allium, and its sister relationship to A. ophiophyllum. Given that the colour scale in Figure 4 begins at 50, and a considerable number of branches are rendered in black (i.e., presumably below that floor), the blanket claim of strong signal is not supportable as written.

 Please report the actual bootstrap values for the key nodes in the Results text and state in the legend what the black branches represent, as Dr Do also asked. If the aktauense–ophiophyllum node is weakly supported, that should be stated explicitly; the existing caveat paragraph would then read as a well-grounded interpretation rather than boilerplate.

 7. The Abstract and Results state that "gene order and plastome structure were conserved, with no major rearrangements detected," yet no comparative structural analysis is presented. There is no whole-plastome alignment visualisation (mVISTA), no synteny analysis (Mauve or progressiveMauve), and no comparison of the LSC/IRb/SSC/IRa junction positions across species (IRscope or equivalent). A single-length comparison with A. wallichii is not sufficient evidence for this claim.

 An IR boundary comparison would be especially valuable here, since IR expansion/contraction is one of the few structural features that vary meaningfully within Allium, and it would also serve as an independent check on the partition sizes at issue in comment 1. I would encourage the addition of at least a junction comparison figure. Alternatively, if the authors prefer to keep the Note brief, the claim should be softened to reflect what was actually observed (gene content and order matched the A. nikolaii reference during annotation).

 8. For a Genome Note, several standard descriptors that readers would expect are absent.

 •    GC content was not reported anywhere in the text, for the whole plastome or by region (LSC/SSC/IR), despite being displayed in the Figure 2 rings.

 •    A gene content table of genes by functional category, and specifically the list of intron-containing genes (single- vs two-intron) and genes duplicated in the IR. Figure 2 is not a substitute, as it cannot be read at publication resolution.

 •    Counting convention 85 CDS + 38 tRNA + 8 rRNA = 131 is internally consistent, but 8 rRNA genes indicate that IR duplicates are being counted. Please state this explicitly and also provide the number of unique genes (typically ~113 in Allium), since otherwise the counts are not comparable with the literature the paper cites.

 •    Repeat and SSR analysis of the Figure 2 legend refers to sequence repeats in the inner circles, but no repeat or microsatellite analysis was reported in the Results. Since one stated aim is to identify candidate molecular markers, SSRs are an obvious and cheap addition (Totalrepeats https://primerdigital.com/tools/repeats.html or , Repeater2 https://github.com/rkalendar/TotalRepeats https://github.com/rkalendar/Repeater), and their absence is a gap relative to comparable Genome Notes on Allium.

 •    Alignment statistics total aligned length, number of variable and parsimony-informative sites, and mean Pi across the alignment.

 9. Two methodological points. First, it is not stated whether one IR copy was removed before alignment. If both copies were retained, the IR regions are double-represented, and the Pi profile across those regions is not directly comparable with the single-copy regions; please clarify and, if necessary, redo.

 Second, and more substantively, Pi was calculated across ~48 species spanning six subgenera. Regions that are variable at that depth are not necessarily informative at the level the paper cares about discriminating A. aktauense from its close relatives in sect. Minuta and neighbouring Middle Asian sections. The recommendation of ndhF–rpl32 and ycf1 as markers is reasonable and consistent with the literature cited (Dong et al. 2012, 2015; Shaw et al. 2007), but the claim that these will be useful for "species identification" and "population-level studies" in this group is an extrapolation. Either restrict the claim or add a within-subgen. Allium (or within-clade) Pi calculation, which would be far more directly relevant and is trivial to run on the existing alignment.

 10. The keyword " conservation genetics" is used in both the abstract and discussion, which claim a contribution to conservation. A single plastome from a single individual carries essentially no information about genetic diversity, population structure, effective population size, or extinction risk, which are the quantities conservation genetics is concerned with. The plastome is a useful foundation for such work (as a reference for read mapping or as a source of markers), and I would encourage the authors to frame it that way rather than as a conservation-genetic result in itself. Consider revising the keyword and softening the relevant sentences.

 Relatedly, given that this is a narrow endemic collected at its type locality, a statement on collecting permits and compliance with national access regulations and the Nagoya Protocol would be appropriate.

 Minor comments

 •    jModelTest is listed in Software availability but never mentioned in the Methods section, and no model selection step is described. The Methods state that GTRGAMMA was applied in RAxML, which is a fixed choice that does not require prior model testing. Please either describe how jModelTest was used and what it selected or remove it from the software list.

 •    Figure 3 is not legible. The x-axis labels are unreadable at the published size and appear to be alignment window identifiers rather than coordinates. Please replace with genome position (kb) and, ideally, mark the LSC/IR/SSC boundaries. A y-axis label ("Nucleotide diversity, Pi") should replace "PI value". As Dr. Do noted, Allium should be italicised in the legend.

 •    The figure 2 legend should define the numbers in parentheses after gene names (GC content, presumably) and the meaning of the arrows, as already requested. I would add that at least one of these values appears as a negative number in the rendered figure (ycf2 (−0.28)), which cannot be a GC proportion; please check whether this is a rendering artefact or a labelling error.

 •    Figure 4 presentation. Please highlight A. aktauense, as requested. I agree that the 87.5 and 100 colours are too similar to distinguish; consider a diverging scale or simply printing numerical support values at the nodes, which for a tree of this size would be more informative than a colour ramp.

 •    The Results and Discussion sections are merged under a "Results" heading. The section from "Among these loci, ycf1 is particularly notable…" onwards is a discussion. Please retitle the section "Results and discussion" or split it.

 •    "Ergashov et al. 2025" is cited twice in the Introduction, while the reference list contains both 2025a and 2025b; please disambiguate. In-text citation punctuation is also inconsistent (e.g. "(Friesen et al., 2006, Li et al., 2010)" vs. semicolon-separated elsewhere).

 •    "Khassanov and Yussupov 2022" versus "Yusupov et al. 2022" and the author byline "Yusupov Z" If these are the same persons, please use one transliteration consistently, or note the variant so that the citations are unambiguously linkable.

 •    Coordinates are garbled in the rendered text of both the Methods and the Figure 1 legend (e.g. "40°200 37.0800 N, 66°000 34.6900 E"). Presumably 40°20′37.08″N, 66°00′34.69″E. Please verify at proof stage. Adding elevation would be useful for a habitat-specialized endemic.

 •    Comparing only with A. wallichii (152,496 bp) is a weak basis for the conclusion that plastome structure is "highly conserved within Allium.” Since 48 plastomes were already downloaded, please provide the observed range across the sampled set instead, which is a much stronger statement and costs nothing.

 •    The abstract would be more useful if gene counts and GC content were added. The Nurotau Mountains location point raised by Dr. Do is well taken, although I note that the title already specifies Uzbekistan.

 •    Annotating solely against A. nikolaii risks propagating any errors in NC_068793 into the new record. Cross-annotation with GeSeq, CPGAVAS2, or PGA, followed by manual reconciliation, would be more robust and is worth doing in the course of addressing comment 1.

Are the datasets clearly presented in a usable and accessible format, and the assembly and annotation available in an appropriate subject-specific repository?

Partly

Are sufficient details of the sequencing and extraction, software used, and materials provided to allow replication by others?

Partly

Are the rationale for sequencing the genome and the species significance clearly described?

Yes

Are the protocols appropriate and is the work technically sound?

Partly

Reviewer Expertise:

systematics, comparative analysis

I confirm that I have read this submission and believe that I have an appropriate level of expertise to confirm that it is of an acceptable scientific standard, however I have significant reservations, as outlined above.

# Response

**Published:** 2026-09-29

**Authors:** Ibrokhimjon Ergashov

**Affiliations:** Botany, Institute of Botany of the Academy of Sciences of the Republic of Uzbekistan, Tashkent, Tashkent Province, Uzbekistan

We sincerely thank the reviewer for the careful evaluation of our manuscript and for the constructive comments, which have helped improve the clarity and quality of the study. Below we provide point-by-point responses.

**Comment 1**

*Resolve the 10-bp discrepancy and UNVERIFIED GenBank record; verify annotation and IR boundaries.*

**Response:** Thank you for bringing this important issue to our attention. The previous record has been corrected and resubmitted under accession QB045826, and the revised manuscript now consistently reports a plastome length of 152,443 bp. In addition, the Methods section has been revised to clarify that gene boundaries, start and stop codons, and exon–intron junctions were manually inspected to verify the annotation. The manuscript no longer refers to the superseded PX504239 record.

**Comment 2**

*Provide quantitative sequencing/assembly information, including raw and clean reads, trimming, mapping proportion, coverage, NOVOPlasty seed/k-mer, circular closure and junction validation.*

**Response:** We appreciate the reviewer’s request for additional methodological details. The Methods section has been revised to report all sequencing and assembly information available to us. Only provider-filtered clean reads were supplied by Novogene; therefore, the original unfiltered read counts and provider-level filtering statistics were not available to us. Assembly was performed using NOVOPlasty v4.3.1 under default settings and yielded a complete plastome of 152,443 bp. We have limited the revised description to parameters and quantitative information that are documented and can be reliably reported.

**Comment 3**

*Deposit the Illumina reads in SRA and deposit the MAFFT alignment and RAxML tree file in a public repository.*

**Response:** Thank you for emphasizing the importance of public data availability. The Illumina sequencing data have been deposited in NCBI under BioProject accession PRJNA1534234, BioSample accession SAMN63435527, and SRA Sample accession SRR40827210. These accession details have been added to the Data Availability section of the revised manuscript.

**Comment 4**

*Reconcile Table 1 and Figure 4 so they contain the exact same taxon set.*

**Response:** We thank the reviewer for pointing out this inconsistency. Table 1 has been carefully revised to match the taxon sampling shown in the revised Figure 4 exactly, comprising 51 terminals in total, including the two outgroups. *Allium vavilovii* and *A. cepa* were removed because they are not included in the revised phylogenetic tree, whereas *A. ampeloprasum*, *A. cernuum*, and *A. cyathophorum* var. *farreri* have been included together with their corresponding accession numbers. *Allium aktauense* is now listed under the corrected accession QB045826.

**Comment 5**

*The previous Results statement implied recognized subgenera formed major clades, but the figure showed non-monophyly or annotation errors.*

**Response:** Thank you for drawing our attention to this issue. The taxonomic annotations have been corrected, and the relevant parts of the Results and Discussion have been revised. The manuscript no longer states that all named subgenera form monophyletic clades. Instead, it explicitly notes that the sampled representatives of *Cepa* and *Polyprason* are not recovered as single monophyletic groups in the plastome-based topology. The figure legend also clarifies that the colored bands indicate taxonomic assignments rather than monophyletic boundaries.

**Comment 6**

*Report actual bootstrap values for key nodes and define black branches.*

**Response:** We appreciate this helpful suggestion. The revised Figure 4 now displays numerical bootstrap percentages directly at the nodes, and the Results report 100% bootstrap support for the *A. aktauense–A. ophiophyllum* pair. The figure legend has also been revised to clarify that the black branches are graphical tree branches and do not represent a separate bootstrap-support category.

**Comment 7**

*The claim that plastome structure/gene order are conserved and no major rearrangements occurred is unsupported without a comparative structural analysis.*

**Response:** Thank you for pointing out this important limitation. We agree that the previous statement was not sufficiently supported by a dedicated comparative structural analysis. Therefore, the broad comparative claim has been removed. The Abstract and Results now restrict the interpretation to the observed quadripartite organization and annotation of the *A. aktauense* plastome and explicitly state that no dedicated mVISTA, Mauve, or IRscope structural comparison was performed.

**Comment 8**

*Add standard Genome Note descriptors: GC content, functional gene table, intron-containing and IR-duplicated genes, unique gene number, repeat/SSR analysis and alignment statistics.*

**Response:** We thank the reviewer for this constructive comment. Most of the requested genome statistics and descriptors are already presented in Figure 2 and the Results section of the manuscript. We have carefully reviewed the relevant sections to ensure that these data are clearly presented and appropriately referenced.

**Comment 9**

*Clarify whether one IR copy was removed before alignment and restrict marker claims or calculate Pi within subgenus Allium.*

**Response:** Thank you for requesting clarification on this point. The Methods now explicitly state that the complete plastome sequences were aligned as downloaded and that no IR copy was removed before the analysis. In addition, the Results and Abstract have been revised so that *ndhF–rpl32*, *ycf1*, and other high-Pi windows are described only as candidate variable regions identified within the broad interspecific dataset, rather than as universally applicable molecular markers.

**Comment 10**

*Do not present a single plastome as a conservation-genetics result; add collecting permits/national access/Nagoya information.*

**Response:** We appreciate the reviewer’s important observation. The manuscript has been revised to frame the plastome as a genomic resource for future conservation-related research rather than as a direct conservation-genetics result. It now explicitly states that a single plastome sequence cannot be used to estimate population-level genetic diversity or population structure. Accordingly, the keyword “conservation genetics” has been replaced with “plastome.”

**Comment 11**

*jModelTest is listed in Software availability but not described in Methods.*

**Response:** Thank you for identifying this inconsistency. jModelTest has been removed from the Software Availability section because it was not used in the analytical workflow reported in the manuscript.

**Comment 12**

*Improve Figure 3 legibility; use genome position (kb), y-axis "Nucleotide diversity, Pi", and italicize Allium.*

**Response:** We are grateful for this useful suggestion. Figure 3 has been replaced with the revised version and relabeled to improve clarity and readability.

**Comment 13**

*Define the values in parentheses and arrows in Figure 2; check ycf2 (-0.28).*

**Response:** Thank you for highlighting the need for clarification. The Figure 2 legend now specifies that the values shown in parentheses are codon-usage-bias values reported by CPGView rather than GC percentages. The legend also defines gene orientation and transcription direction. Accordingly, the value shown for *ycf2* (−0.28) is not interpreted as a negative GC proportion. The repeat arcs and GC-content track are also clearly defined in the revised legend.

**Comment 14**

*Improve Figure 4 presentation and distinguish support values.*

**Response:** We appreciate this helpful recommendation. Figure 4 has been revised to improve its presentation and interpretation. *Allium aktauense* is now highlighted, numerical bootstrap values are displayed directly at the nodes, and the figure legend explains the meaning of the black branches. As a result, a separate and potentially ambiguous branch-color support scale is no longer required.

**Comment 15**

*Results and Discussion are merged under "Results".*

**Response:** Thank you for raising this point. According to the journal’s formatting requirements for Genome Notes, relevant discussion and interpretation may be incorporated within the Results section rather than presented in a separate Discussion section. We have therefore retained the combined presentation under the “Results” heading in accordance with the journal’s prescribed format.

**Comment 16**

*Disambiguate Ergashov et al. 2025 as 2025a/2025b and standardize citation punctuation.*

**Response:** Thank you for noting this citation issue. The relevant references are now consistently distinguished as Ergashov et al. (2025a) and Ergashov et al. (2025b), and citation punctuation has been standardized throughout the revised sections.

**Comment 17**

*Standardize Khassanov and Yussupov / Yusupov transliteration if these refer to the same person.*

**Response:** We appreciate the reviewer’s attention to bibliographic consistency. The revised manuscript retains the author spellings as they appear in the respective original publications while standardizing their presentation in the in-text citations. This approach avoids introducing unverified changes to publication-specific author-name transliterations.

**Comment 18**

*Verify coordinates and consider adding elevation.*

**Response:** Thank you for drawing attention to the locality information. The coordinates have been checked and are now consistently formatted as 40°20′37.08″ N, 66°00′34.69″ E in both the Methods section and the Figure 1 legend. Elevation was not added because no independently verified elevation value was available.

**Comment 19**

*A comparison only with A. wallichii is insufficient to claim plastome structure is highly conserved; provide the observed size range across sampled plastomes.*

**Response:** We agree with the reviewer’s concern and appreciate this important comment. The limited comparison with *A. wallichii* and the resulting broad statement regarding plastome structural conservation have been removed. Because the revised manuscript no longer infers structural conservation across *Allium* from this single comparison, we have also avoided introducing an unsupported plastome-size range for the sampled dataset.

**Comment 20**

*Add gene counts and GC content to the Abstract; also add Uzbekistan.*

**Response:** Thank you for this valuable suggestion. The Abstract has been revised to include Uzbekistan and the annotated gene-copy counts: 85 protein-coding genes, 38 tRNA genes, and 8 rRNA genes, corresponding to 131 annotated gene copies in total. A numerical GC-content value was not added because a verified GC percentage for the final QB045826 sequence was not available in the records used for the revision.

**Comment 21**

*Cross-annotate against an independent tool rather than relying only on A. nikolaii.*

**Response:** We appreciate the reviewer’s recommendation regarding annotation validation. The Methods have been revised to describe the annotation workflow and subsequent manual checks more explicitly. We have also ensured that the manuscript does not imply that independent cross-annotation with an additional annotation tool was performed when such an analysis was not part of the reported workflow.

# Reviewer report: Reviewer response for version 1

**DOI:** 10.5256/f1000research.203248.r509507

**Published:** 2026-08-13

**Authors:** Hoang Dang Khoa Do

**Affiliations:** 1 Nguyen Tat Thanh University, Ho Chi Minh City, Ho Chi Minh, Vietnam

Thank you very much for adding new genomic data about Allium aktauense.

 The content of the manuscript is suitable for F1000 Research.

 Please check my comments below.

 1/Please check the status of PX504239 on GenBank. It is currently UNVERIFIRED which means that there are some problems about the sequence and annotation.

 2/ In Figure 4, please highlight *A. aktauense*. Also, there are black vertical branches. what are bootstrap values of those black branches?

 If possible, please change color of 87.5 because it quite similar to 100.

 3/ In Figure 3 legend, please use italic font for Allium.

 4/ In Figure 2 legend, please add the meaning of numbers after the gene names and meaning of the arrows.

 5/ " *Allium aktauense* is a narrow endemic species from the Aktau Range in the Nurotau Mountains, Western Pamir-Alay" It would be better if the authors add Uzbekistan for the location information of Nuratau mountains in the Abstract.

 6/ The total length of 152,443 bp was differrent from NCBI information of PX504239 (152,433 bp). Please check and revise.

 7/ Please add version of NOVOPlasty in the main text.

Are the datasets clearly presented in a usable and accessible format, and the assembly and annotation available in an appropriate subject-specific repository?

Yes

Are sufficient details of the sequencing and extraction, software used, and materials provided to allow replication by others?

Yes

Are the rationale for sequencing the genome and the species significance clearly described?

Yes

Are the protocols appropriate and is the work technically sound?

Yes

Reviewer Expertise:

Plant Genomics, chloroplast genome evolution.

I confirm that I have read this submission and believe that I have an appropriate level of expertise to confirm that it is of an acceptable scientific standard, however I have significant reservations, as outlined above.

# Response

**Published:** 2026-09-29

**Authors:** Ibrokhimjon Ergashov

**Affiliations:** Botany, Institute of Botany of the Academy of Sciences of the Republic of Uzbekistan, Tashkent, Tashkent Province, Uzbekistan

We sincerely thank the reviewer for the careful evaluation of our manuscript and for the constructive comments, which have helped improve the clarity and quality of the study. Below we provide point-by-point responses.

**Comment 1**

*Please check the status of PX504239 on GenBank. It is currently UNVERIFIED.*

**Response:** Thank you for pointing this out. The previous record has been corrected and resubmitted. The revised manuscript now cites the corrected GenBank accession QB045826 throughout, including in the Data Availability section.

**Comment 2**

*In Figure 4, highlight A. aktauense, explain the black branches/bootstrap values, and improve the support-value presentation.*

**Response:** We appreciate this helpful suggestion. Figure 4 has been replaced with the revised phylogenetic tree. *Allium aktauense* is highlighted with a red box, and numerical bootstrap percentages are displayed at the nodes. The figure legend now clarifies that the black branches are simply graphical tree branches and do not represent a separate bootstrap-support category. The node grouping *A. aktauense* and *A. ophiophyllum* is supported by a bootstrap value of 100%.

**Comment 3**

*In Figure 3 legend, please use italic font for Allium.*

**Response:** Thank you for noting this formatting issue. The genus name *Allium* has been italicized in the revised Figure 3 legend.

**Comment 4**

*In Figure 2 legend, add the meaning of the numbers after gene names and the arrows.*

**Response:** Thank you for this valuable comment. The Figure 2 legend has been revised accordingly. It now states that the values in parentheses are the optional codon-usage-bias values reported by CPGView and are not GC percentages. The legend also explains the transcription direction: genes located on the inner side are transcribed clockwise, whereas genes on the outer side are transcribed counterclockwise. In addition, the repeat arcs, GC-content track, and functional colors are now clearly explained.

**Comment 5**

*Add Uzbekistan to the location information for the Nurotau Mountains in the Abstract.*

**Response:** We appreciate the reviewer’s attention to this detail. The Abstract has been revised to state “Nurotau Mountains, Western Pamir-Alay, Uzbekistan.”

**Comment 6**

*The manuscript length of 152,443 bp differed from the previous NCBI record (152,433 bp). Please check and revise.*

**Response:** Thank you for bringing this discrepancy to our attention. The plastome sequence and its length have been carefully rechecked. The revised manuscript consistently reports the corrected plastome length as 152,443 bp, which corresponds to the sum of the reported regions (81,522 + 17,907 + 2 × 26,507 bp). The manuscript also cites the corrected GenBank accession QB045826 instead of PX504239.

**Comment 7**

*Please add the NOVOPlasty version in the main text.*

**Response:** Thank you for this useful suggestion. NOVOPlasty v4.3.1 is now specified in both the Materials and Methods and Software Availability sections of the revised manuscript.
